# [Expression of Concern] miR-221/222 promote malignant progression of glioma through activation of the Akt pathway

**DOI:** 10.3892/ijo.2025.5808

**Published:** 2025-10-09

**Authors:** Junxia Zhang, Lei Han, Youlin Ge, Xuan Zhou, Anling Zhang, Chunzhi Zhang, Yue Zhong, Yongping You, Peiyu Pu, Chunsheng Kang

Int J Oncol 36: 913-920, 2010; DOI: 10.3892/ijo_00000570

Following the publication of the above paper, a concerned reader drew to the Editor's attention that, for the immunohistochemistry images shown in Fig. 6, the Control/PCNA and Control/p27kip1 panels appeared to be duplicates of each other, where the results of differently performed experiments were intended to have been portrayed.

The authors were contacted by the Editorial Office to offer an explanation for this potential anomaly in the presentation of the data in this paper, although up to this time, no response from them has been forthcoming. Owing to the fact that the Editorial Office has been made aware of potential issues surrounding the scientific integrity of this paper, we are issuing an Expression of Concern to notify readers of this potential problem while the Editorial Office continues to investigate this matter further.

